# Extracellular vesicles as distinct biomarker reservoirs for mild traumatic brain injury diagnosis

**DOI:** 10.1093/braincomms/fcab151

**Published:** 2021-07-08

**Authors:** Kryshawna Beard, Zijian Yang, Margalit Haber, Miranda Flamholz, Ramon Diaz-Arrastia, Danielle Sandsmark, David F Meaney, David Issadore

**Affiliations:** 1 Department of Pharmacology, Perelman School of Medicine, University of Pennsylvania, Philadelphia, PA 19104, USA; 2 Department of Mechanical Engineering and Applied Mechanics, University of Pennsylvania, Philadelphia, PA 19104, USA; 3 Department of Neurology, University of Pennsylvania, Philadelphia, PA 19104, USA; 4 Department of Bioengineering, University of Pennsylvania, Philadelphia, PA 19104, USA; 5 Department of Neurosurgery, University of Pennsylvania Perelman School of Medicine, Philadelphia, PA 19104, USA; 6 Department of Electrical and Systems Engineering, University of Pennsylvania, Philadelphia, PA 19104, USA

**Keywords:** traumatic brain injury, biomarkers, extracellular vesicles, machine learning

## Abstract

Mild traumatic brain injury does not currently have a clear molecular diagnostic panel to either confirm the injury or to guide its treatment. Current biomarkers for traumatic brain injury rely mainly on detecting circulating proteins in blood that are associated with degenerating neurons, which are less common in mild traumatic brain injury, or with broad inflammatory cascades which are produced in multiple tissues and are thus not brain specific. To address this issue, we conducted an observational cohort study designed to measure a protein panel in two compartments—plasma and brain-derived extracellular vesicles—with the following hypotheses: (i) each compartment provides independent diagnostic information and (ii) algorithmically combining these compartments accurately classifies clinical mild traumatic brain injury. We evaluated this hypothesis using plasma samples from mild (Glasgow coma scale scores 13–15) traumatic brain injury patients (*n *=* *47) and healthy and orthopaedic control subjects (*n *=* *46) to evaluate biomarkers in brain-derived extracellular vesicles and plasma. We used our Track Etched Magnetic Nanopore technology to isolate brain-derived extracellular vesicles from plasma based on their expression of GluR2, combined with the ultrasensitive digital enzyme-linked immunosorbent assay technique, Single-Molecule Array. We quantified extracellular vesicle-packaged and plasma levels of biomarkers associated with two categories of traumatic brain injury pathology: neurodegeneration and neuronal/glial damage (ubiquitin C-terminal hydrolase L1, glial fibrillary acid protein, neurofilament light and Tau) and inflammation (interleukin-6, interleukin-10 and tumour necrosis factor alpha). We found that GluR2+ extracellular vesicles have distinct biomarker distributions than those present in the plasma. As a proof of concept, we showed that using a panel of biomarkers comprised of both plasma and GluR2+ extracellular vesicles, injured patients could be accurately classified versus non-injured patients.

## Graphical Abstract

**Graphical Abstract:**
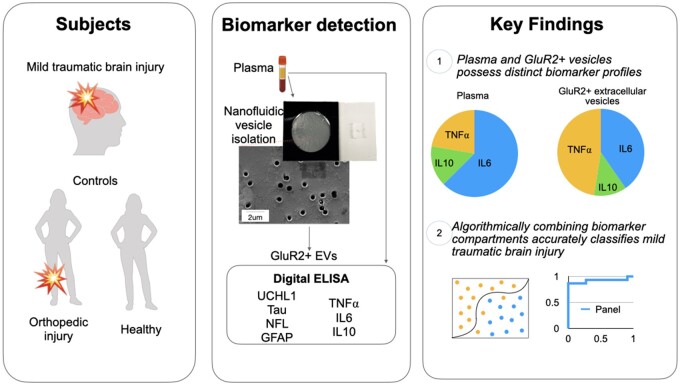

## Teaser

Abbreviated summary

Beard et al. report that circulating brain-derived extracellular vesicles captured using a novel nanofluidic device contain distinct molecular information from conventional plasma biomarkers of traumatic brain injury. They demonstrate that algorithmically combining biomarkers from both compartments may provide a clinically useful, information-rich method of classifying brain injury.

## Introduction

Although most individuals who experience a mild traumatic brain injury (mTBI) recover within weeks after the injury, a significant number of patients suffer from persistent symptoms that include headaches, cognitive changes and mood disturbances for months afterward.^1–3^ Conventional approaches to evaluate mTBI have focussed on currently known hallmarks of moderate-to-severe brain damage, such as clinical assessment using the Glasgow Coma Scale, macroscale lesions visualized with CT imaging and circulating neurodegenerative markers.^4^ Unfortunately, these methods lack the sensitivity and specificity needed to clinically characterize milder injuries, to identify patients who are likely to have persistent symptoms in the time following mTBI, and to guide each patient to a personalized, effective therapy.^5^ Because adequate biomarkers are lacking, the identification of mTBI patients in need of intervention remains mainly limited to monitoring for and treating post-concussive symptoms as they arise rather than treating the underlying pathology much earlier in the course of the disease, when therapies are more likely to be effective.

Biomarker discovery to accurately diagnose and classify an individual’s TBI into categories for improving individual patient outcomes and developing new treatments has generated great interest in recent years. However, identifying sufficiently sensitive and specific biomarkers has been confounded by the particularly dynamic and heterogeneous nature of TBI. Each TBI results in a unique combination of initial tissue damage and secondary pathology including vascular dysfunction,^6^^,^^7^ axonal injury^8^^,^^9^ and inflammation^10^ that evolve following the injury.^11–13^ These distinct aspects of an individual’s TBI—each of which maps to multiple potential biomarkers in the blood—is considered key to a patient’s possible recovery or progression to behavioural and cognitive deficits.^14^^,^^15^ Moreover, profiles of biomarkers in the blood are dynamic, originating from both the initial tissue damage and the multiple secondary pathologies that develop over time after the injury.^14^^,^^16^^,^^17^ The complexity of biomarker expression following an injury results in diagnostics measurements that can be challenging to interpret.

To gain a more comprehensive assessment of mTBI, many researchers have shifted their attention away from measurements of single biomarkers to measurements of biomarker panels, where each constituent biomarker can be chosen to assess a different aspect of the patient's TBI.^18^ For example, combined analysis of circulating glial fibrillary acidic protein (GFAP), an astrocyte-derived intermediate filament protein, and ubiquitin C-terminal hydrolase L1 (UCHL1), a neuronal cytosolic protein, accurately identifies injury severity and CT scan lesions in clinical TBI. While this assay—the Banyan Brain trauma indicator test—has demonstrated promise as a TBI diagnostic for more severe injuries, such biomarkers have not yet been identified that reliably classify underlying TBI endophenotypes or predict patient outcomes after mTBI.^19^ Other proposed TBI biomarkers have potential to directly assess specific underlying TBI pathologies. These include neurofilaments,^20^^,^^21^ a major cytoskeletal component of neuronal axons, and Tau, a cytoskeletal protein whose phosphorylation and aggregation are hallmarks of neurodegenerative conditions.^22^^,^^23^ Dysregulated central and peripheral immune cell function following TBI results in the release of cytokines, chemokines and complement components that may provide an assessment of inflammation, a key driver of neurologic deficit post-TBI.^24^^,^^25^

Extracellular vesicles (EVs) have generated particular interest for multiplexed TBI diagnostics. EVs are nanoscale vesicles ranging from 100 to 1000 nm^26^ formed through a variety of mechanisms including plasma membrane budding or the fusion of multivesicular bodies (MVBs) to the cellular membrane for release into the extracellular space.^27^ EVs possess surface proteins derived from the parent cell, and cargo (proteins, mRNA, miRNAs) within the vesicle lumen that reflect the status of their cells of origin, and that, when transferred to recipient cells, can act as agents of cell–cell communication.^28–30^ EVs are emerging as a promising complement to plasma-derived biomarkers, as they contain cargo that may play direct roles in TBI pathology, and contain surface proteins that allow brain-derived EVs to be isolated from the blood. EVs and their cargo also provide a work-around to the impracticality of brain tissue biopsy by crossing the blood–brain barrier into CSF,^31^ peripheral circulation,^32^ and other bodily fluids making them easily accessible CNS biomarkers for monitoring TBI progression.^33^^,^^34^ Moreover, EVs are shed by both healthy and degenerating cells, providing a broader view into the molecular processes that occur within a tissue or organ. The combination of information extracted from the EVs of injured, but not necessarily degenerating, neurons with neuronal biomarkers and inflammatory mediators could lead to accurate classifications and prognoses of patients with mTBI.

On their own, the diagnostic potential of EVs has been shown in military personnel, where circulating exosome-packaged Tau and IL10 levels are elevated with mTBI and correlate with post-concussive and post-traumatic stress disorder symptoms.^35^ Other studies have found variations in EV microRNA concentration after TBI.^36^ In previous work, we showed that by enriching brain associated EVs from plasma, which expressed the glutamate ionotropic receptor AMPA type subunit 2 (GluR2) surface marker, from plasma using a nanomagnetic chip, and analysing RNA cargo we could identify RNA signatures that accurately classified the injury, including its presence, severity, history of previous injuries and timing.^37–39^ However, this work was limited to the RNA cargo of EVs, and did not incorporate known biomarkers of neuronal and glial cell damage or inflammation packaged in EVs.^35^

In this study, we combined conventional assessment of TBI-associated biomarkers in plasma with our approach of acquiring molecular information from brain-derived EVs. Our main purpose was 2-fold: to determine if EVs and plasma biomarker proteins represented *independent* information for mTBI diagnostic use, and to evaluate the relative effectiveness of applying this approach on a set of mTBI patients. We used our TENPO technology to enrich for brain-derived EVs and leveraged an existing ultrasensitive digital enzyme-linked immunosorbent assay (ELISA) technique, Single-Molecule Array (SIMOA), to accurately determine common protein biomarker levels in these two compartments. We demonstrate that the independence of molecular information stored in the GluR2+ EVs and in plasma allows for the development of a multianalyte approach to mTBI diagnosis. In this work, we use a machine learning algorithm developed using biomarkers from both compartments as a proof-of-concept of this approach, and illustrate its ability to successfully discriminate mTBI patients from control subjects.

## Materials and methods

### Study design, participants and sample collection

**Figure 1:**
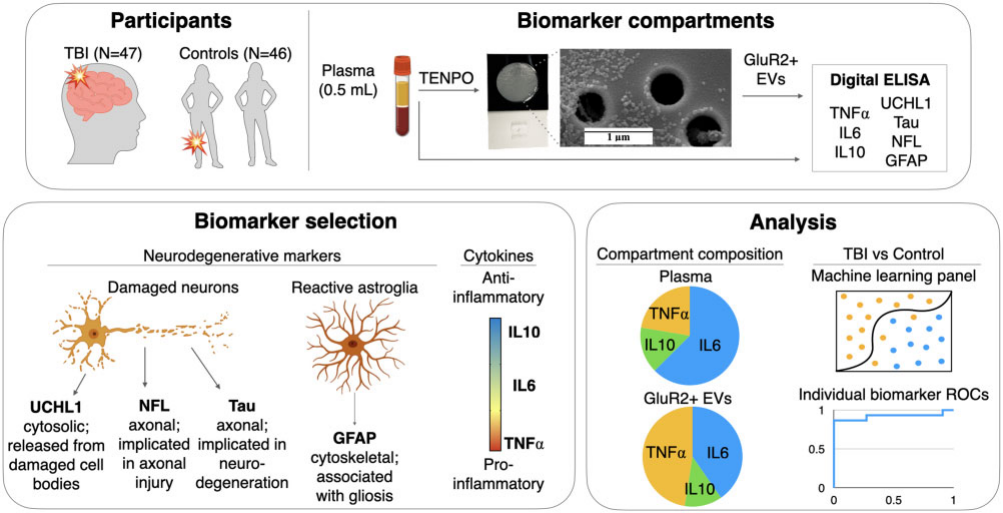
**Project workflow.** (**A**) Samples were obtained from subjects sustaining TBIs through a variety of mechanisms and from a combination of orthopaedic injured and healthy controls. One 500 µl aliquot of plasma from each subject was used to isolate brain-derived EVs based on their expression of GluR2 using our nanofluidic platform, TENPO. Lysate from GluR2+ EVs and a second 500 µl aliquot of plasma were subjected to digital ELISA assessment. (**B**) Biomarkers were selected based on known or emerging role in neuronal (UCHL1, NFL, Tau) or astrocyte (GFAP) pathology, or on their roles in the spectrum of inflammatory function (TNFα, IL6, IL10). (**C**) Analyses served two purposes: comparison of biomarker distribution in plasma and in GluR2+ EVs, and the discrimination of TBI and control subjects. A machine learning approach was used to combine the multiplexed data into biomarker panels for comparison with the performance of individual biomarker ROC curves and panels of biomarkers from each compartment alone.

Owing to the subtle nature of the physical injury in mTBI, we hypothesized that algorithmically combining biomarkers from both brain-derived EVs and plasma could result in more sensitive and specific discrimination of mTBI patients from controls than that of any individual biomarker, or any one biomarker compartment. To test this hypothesis, we obtained human plasma samples from TBI subjects admitted to an urban, academic Level 1 trauma centre (University of Pennsylvania’s Penn Presbyterian Medical Center) following a head impact—representing the diversity of injury types encountered in the clinic, including assault, road traffic incidents, and falls—as well as healthy control and orthopedically injured participants yielding a study size consistent with previous experiments (Fig. 1A).^38^ Our blood-based assessment of mTBI included a panel of neuronal and glial cell damage biomarkers [UCHL1, neurofilament light (NFL), Tau and GFAP] and key drivers of inflammation [interleukin-6 (IL6), interleukin-10 (IL10) and tumour necrosis factor alpha (TNFα)] quantified in both plasma and within brain-derived EVs expressing GluR2 (Fig. 1B). We then used these data to investigate protein distribution across the two compartments and to evaluate mTBI-associated changes in biomarker concentration and signatures (Fig. 1C).

The protocol to obtain participant samples was approved by the University of Pennsylvania Institutional Review Board. Enrolment began in 2017, and enrolled TBI participants had (i) high clinical suspicion of non-penetrating acute TBI, determined by the treating physicians for which a head CT scan was performed; (ii) age ≥18 years; (iii) interval between time of injury and enrolment <24 h; (iv) admission to the hospital; and (v) the ability to obtain informed consent from the subject or a legally authorized representative. All TBI subjects sustained mild TBIs, as defined by a Glasgow Coma Scale score of 13–15. Healthy control participants (*n *=* *39) had no history of concussion/TBI. Orthopedically injured participants (*n *=* *7) were screened for head impact at the time of their injury and were excluded if they had a head impact or prior history of concussion/TBI. Participants were excluded if they had a history of pre-existing serious neurologic or psychiatric disease that would interfere with outcome assessment, penetrating traumatic brain injury, positive pregnancy test or known pregnancy, or were incarcerated. For this study, we analysed plasma samples collected <24 h from injury prepared from whole blood by centrifugation (1000 rpm for 10 min). Plasma was processed within 1 h of collection, aliquoted at 500 µl, and frozen at −80°C until further analyses.

### TENPO device assembly and vesicle capture and lysis

Isolation of brain-derived EVs was done using our previously reported nanofluidic platform, TENPO.^37–39^ Conventional methods for EV isolation from clinical samples rely on a two-step process: size-based capture followed by immunoprecipitation. Size-based isolation is time consuming, results in impure samples contaminated with cellular debris and loss of the targeted EVs, and must be combined with downstream immunoprecipitation to isolate EVs of specific cellular origins. TENPO overcomes these limitations through its nanoscale immunomagnetic sorting of individual EVs, which renders each labelled EV distinct from background non-specifically labelled EVs.^37–39^ TENPO eliminates the need for a size-based isolation step, vastly reduces the time required to process samples, and results in higher EV recovery from each sample than other isolation methods.^37^ EVs isolated with the same GluR2+ TENPO assay we use in this work were visualized with scanning electron microscopy (repeated for this study in Supplementary Fig. 1), probed for canonical EV markers, and validated with isotype control antibody in accordance with standards devised by the International Society of Extracellular Vesicles^40^ during the assay’s initial development.^37–39^

In previous work, we successfully used TENPO to isolate brain-derived EVs based on their expression of GluR2 from plasma obtained from mouse models of TBI and human clinical TBI patients.^38^ The device consists of track-etched polycarbonate membranes with 600 nm pores (Whatman, Millipore Sigma) coated with a 200 nm layer of permalloy (Ni_80_Fe_20_) and 30 nm layer of gold (Kurt Lesker PVD-75; Singh Nanofabrication Facility, University of Pennsylvania) embedded in layers of moisture-resistant polyester film (McMaster-Carr, 0.004″ thick, Elmhurst, Illinois) and solvent-resistant tape (McMaster-Carr, Elmhurst, Illinois). The reservoir for inputting the sample was laser cut from a cast acrylic sheet (McMaster-Carr), and TENPO’s polydimethylsiloxane output was connected to a negative pressure supply (Programmable Syringe Pump; Braintree Scientific, Braintree, Massachusetts). To isolate brain-derived EVs from plasma, we incubated 500 µl of subject plasma, first with biotin anti-human, mouse GluR1/GluR2 antibody (Bioss, Woburn, Massachusetts) and then with anti-biotin ultrapure microbeads (Miltenyi Biotec, Germany) at room temperature with shaking, each for 20 min. After incubation, we loaded each sample into the reservoir of a TENPO device and ran it through the chip using a programmable syringe pump (Braintree). Captured EVs were lysed using 500 µl 1% SDS in PBS as this completely solubilizes even membrane-bound proteins,^41^ inactivates most cellular proteases,^42^ and is more concentrated than the 0.025% minimum shown to achieve vesicle lysis.^43^ We also found this lysis condition yielded higher protein levels than lysis with RIPA buffer containing 1% SDS (Supplementary Fig. 2). We diluted the lysate 1:10 to reduce the SDS concentration to 0.1% before running the assay. Lysates were stored at −80ºC until dilution and further analysis. To avoid measurement biases, a combination TBI and control subject plasma EVs were obtained during each run.

### Single-Molecule Array digital ELISA measurement

EV lysates and plasma samples were analysed using the Neurology 4Plex (A) and Cytokine 3Plex (A) Single-Molecule Array kits (SIMOA; Quanterix, Billerica, Massachusetts). Five hundred microlitre of plasma samples and quality controls provided by the kit were thawed on ice and spun at 10 000 RCF for 5 min. Supernatant from this spin was used for plasma analysis run using a 1:4 dilution. GluR2+ EV lysates were diluted in the Quanterix-provided sample diluent at a 1:10 ratio. For Neurology 4Plex analysis, 35 µl of EV lysate was combined with 315 µl of Neurology 4Plex sample diluent. For cytokine analysis, 25 µl of EV lysate was combined with 225 µl of Cytokine 3Plex sample diluent. 110–120 µl of plasma and quality control samples were loaded into sample plates and run at 4× dilution. The full volume (315 or 225 µl) of diluted the EV lysate was loaded into sample plates and run neat. The results were corrected using the 10× or 4× dilution factor after analysis. To avoid measurement biases, GluR2+ EV samples and plasma were run on the same plate with their respective SIMOA run settings.

### Statistical analysis of single biomarkers

**Figure 2:**
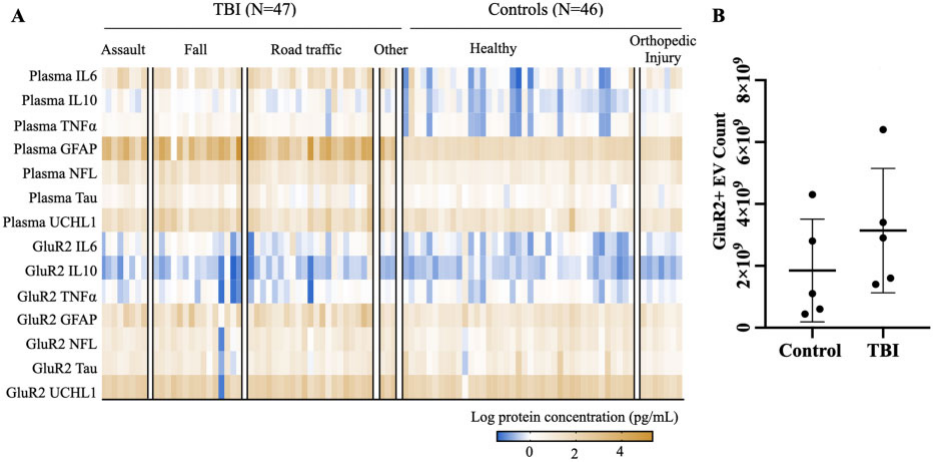
**Expression of brain-derived proteins and cytokines is heterogeneous across TBI and controls in both plasma and GluR2+ EV compartments.** (**A**) Log-transformed biomarker levels plotted in heat map. Columns represent subjects, each arranged within respective TBI or control types by increasing age. (**B**) Number of GluR2+ EVs isolated from 0.5 ml plasma from *N *=* *5 TBI and *N *=* *5 control subjects.

**Figure 3:**
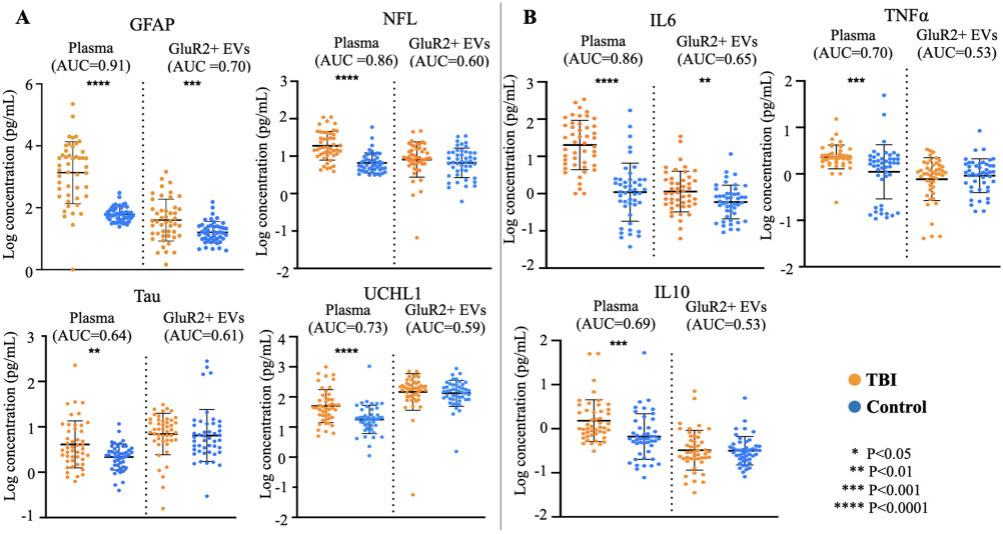
**Mild TBI is associated with elevations in both brain-derived proteins and cytokines in plasma and GluR2+ EVs.** Scatter plots of mean log biomarker values and standard deviation as error bars. Calculation of *P*-values using student’s *t*-test were done using log-transformed data. AUCs were generated using raw values.

We began our analysis by comparing biomarker levels between mTBI and control subjects, and assessing their ability to discriminate the two groups. We used one-way ANOVA (95% confidence intervals) to assess the effects of specific injury/control groups on biomarker expression, and evaluated the correlation between biomarker levels and age. To visualize the spread of the data across individuals, we used the log-transformed values of biomarker measurements to plot heatmaps (Fig. 2) and scatter plots (Fig. 3). For scatter plots, *P*-values were calculated using Student’s *t*-test with log-transformed data. Correlations between biomarkers were calculated using Pearson correlation. To assess each biomarker’s ability to discriminate mTBI from control groups, we calculated receiver operating characteristic (ROC) curves and reported the area under the curve (AUC) for each. To statistically compare the performance of each biomarker, we quantified the standard deviation for each biomarker’s AUC calculation using bootstrapping, analysing a random subset (90% of the total sample size) 10 times with replacement. We compared the AUCs of each individual biomarker with our machine learning model’s AUC via Student’s *t*-test function while taking into account the correlation between the AUCs that is induced by the paired nature of data.^44^ To account for variability in biomarker yield from TENPO-isolated EVs and centrifuged plasma, we reported biomarker expression as the relative abundance of each biomarker in its given category (cytokine or brain-derived protein). To this end, we summed the patient or control average pg/ml levels of plasma IL6, IL10 and TNFα and then divided the pg/ml level of each individual cytokine by this total. We repeated this process with EV-packaged biomarkers, and for the brain-derived proteins.

### Machine learning analysis

We used a machine learning-based approach to algorithmically determine a biomarker signature that discriminates mTBI from control subjects, using protein concentrations from both plasma and GluR2+ EVs. Our machine learning approach consisted of two stages. In the first, we used one set of patient (*n *=* *30) and control (*n *=* *31) samples for feature selection and model training. To perform feature selection and train our model, we first performed Least Absolute Shrinkage and Selection Operator (LASSO) to attain a biomarker panel. To mitigate the effect of overfitting, we averaged the predictive values of an ensemble of five classifier algorithms—K-Nearest-Neighbors, SVM, Linear Discriminate Analysis, Logistic Regression and Naive Bayes—instead of relying on a single classifier.^41^ Additionally, we applied a bootstrapping method that averages the predictions of multiple subgroups of the training set to diminish the effects of outlier data. We performed initial evaluation of the performance of the machine learning panels using 5-fold cross validation. In the second stage of our machine learning analysis, we validated the model developed in training phase with a user-blind, independent set of patient (*n *=* *15) and control (*n *=* *11) subjects. Testing was done only once to avoid the possibility of overfitting our model to the test set data. The classifier model was implemented in Python and LASSO was carried out using standard machine learning packages in Matlab 2017a. Of the 93 total subjects assessed, 61 were used during machine learning training. Of the 32 subjects later obtained during user-blind assessment of our machine learning algorithm, 6 were excluded for incomplete biomarker data.

To assess the performance of our multi-analyte approach, we also generated biomarker panels obtained from plasma brain-derived proteins, plasma cytokines, GluR2+ EV brain-derived proteins and GluR2+ EV cytokines. For statistical analysis, we randomly selected 90% of the total test set to evaluate the variances of our prediction, and repeated this approach 10 times to obtain an average AUC and standard deviation across panels. We compared the average AUC from each panel with our original machine learning panel using *t*-tests while taking into account the correlation between AUCs that is induced by the paired nature of data.^44^

### Scanning electron microscopy

We imaged captured GluR2+ EVs by fixing them to the TENPO membrane using 2.5% glutaraldehyde, 2.0% paraformaldehyde in 0.1 sodium cacodylate buffer, pH 7.4. Images were taken at the Cell and Developmental Biology Microscopy Core at University of Pennsylvania.

### Nanotracking analysis

500 µl of plasma from 5 TBI and 5 control subjects was used to isolate GluR2+ vesicles with TENPO, and eluted in 500 µl of PBS. Vesicles were diluted 1:1000 in molecular grade water. Nanotracking analysis was performed at the Extracellular Vesicle Core facility at University of Pennsylvania and particle count results were corrected with the dilution factor. Student’s *t*-test was used to assess differences in particle count across the groups.

### Data availability

Data were generated at the School of Engineering and Applied Science at the University of Pennsylvania and Penn Presbyterian Medical Center. Data supporting the findings of this study are available from the corresponding author upon request.

## Results

### Participant demographics

To test our hypothesis that algorithmic combination of biomarker data yields a more accurate mTBI diagnostic, we collected plasma and EV samples from GCS mild (13–15) clinical TBI patients (*n *=* *47; Fig. 1). The study design also included a control group (*n *=* *46) consisting of both healthy age-matched and orthopaedic injured controls to assess the specificity of blood and EV-packaged biomarkers to mTBI. Although controls and mTBI subjects were of similar ages (mean = 36 years ± 16 TBI, ±14 controls), there were 20% more males in the mTBI than in the control group (Table 1).

**Table 1:** Descriptive characteristics of traumatic brain injury patient and control subjects; mean ± SD or *N* (%).

Characteristics	TBI	Control	TBI	Control
Machine learning group	Training	Training	Test	Test
*N*	30	31	17	15
Demographics				
Age, mean ± SD (years)	35 ± 14	36 ± 16	44 ± 18	28 ± 8
Male gender, *N* (%)	83	53	61	60
GCS mean	14.4	N/A	14.5	N/A
Positive CT, *N* (%)	57	N/A	72	N/A

### Plasma- and brain-derived GluR2+ vesicles display variable biomarker distribution across individuals

To visualize the spread of the data across individuals, we first plotted log values of each biomarker across TBI and control subjects (Fig. 2A). For the mTBI group, coefficient of variance (CV) values for plasma biomarker levels ranged from 31% for GFAP, to 120% for TNFα. In the control subjects, CV values for plasma biomarkers ranged from 27% for IL10, and 140% for GFAP. Levels of plasma biomarkers for mTBI subjects were not significantly affected by injury type (Supplementary Table 1; ANOVA; *P* > 0.65 across all biomarkers), nor did they correlate with age (Supplementary Table 5). We thus collapsed all injury types into a single mTBI group for our subsequent analysis of plasma biomarker measures. In the control group, orthopaedic controls exhibited significantly higher levels of plasma GFAP compared to the control mean (Supplementary Table 2; ANOVA; *P* < 0.05; Dunett correction for multiple comparisons), but there were no additional effects of control type or age on plasma biomarker levels. We thus consolidated control subjects into a single group for plasma biomarker analyses.

Our analysis and visualization of the data also revealed, similar to plasma biomarkers, that proteins packaged in GluR2+ EVs are also expressed heterogeneously across individual subjects (Fig. 2A). For the mTBI group, CV for GluR2+ EV-packaged biomarkers ranged from 45% for IL6 to 140% for Tau. We found neither injury/control type nor age has an effect on the expression of EV-packaged biomarkers (Supplementary Tables 3–4; *P* > 0.38 (injury type, across all biomarkers); *P* > 0.24 (control type, across all biomarkers)). Nor were there any significant correlations between age and GluR2+ EV biomarker levels for either group (Supplementary Table 6; *P* > 0.05 across all biomarkers for both groups). Therefore, we consolidated all injury types into a single injury group, and the two control types into a second group for analyses of these biomarkers. Furthermore, we demonstrated that there is no significant difference in the number of EVs across TBI and controls (*P* > 0.05), eliminating the need to normalize across EV count (Fig. 2B).

Once we consolidated our dataset, we hypothesized—based on other studies of mTBI biomarkers, and on the rate of CT scan abnormality of our mTBI subjects (Table 1)—that mTBI subjects would exhibit significant elevations in conventionally-studied plasma biomarkers relative to controls.^19^ To test this hypothesis, and to investigate whether mTBI was also associated with significant changes to biomarker levels in the GluR2+ EV compartment, we compared mean levels of individual biomarkers in both compartments (Fig. 3).

Of the seven biomarkers measured, GFAP and IL6 were each significantly elevated in both plasma (GFAP *P* < 0.0001, 147-fold change compared to controls; IL6 *P* < 0.01, 7-fold change relative to controls) and GluR2+ EVs (GFAP *P* < 0.001, 6-fold change compared to controls; IL6 *P* < 0.01, 3-fold change relative to controls) in mTBI. Of the remaining brain-derived proteins, plasma levels of NFL, Tau and UCHL1 were all significantly elevated in the mTBI group. (NFL *P* < 0.0001, 3-fold change relative to controls; Tau *P* < 0.01, 4-fold change relative to controls; UCHL1 *P* < 0.0001, 3-fold change relative to controls). GluR2+ EV levels of NFL, Tau and UCHL1 were unchanged following mTBI. Of the two remaining cytokines, both were significantly elevated in plasma in the mTBI group (IL10 *P* < 0.001, 2-fold change relative to controls; TNFα *P* < 0.001, 1-fold change relative to controls). Neither of these cytokines were significantly altered in the GluR2+ EV compartment following mTBI. Additionally, biomarkers significantly elevated in mTBI plasma also displayed the highest AUCs for discriminating mTBI and control subjects (plasma GFAP = 0.91; GluR2+ EV GFAP = 0.70; plasma NFL = 0.86; plasma IL6 = 0.86). AUCs for all other biomarkers were likely affected by the heterogeneous distribution of the dataset.

### Brain-derived EVs and plasma possess distinct protein compositions

**Figure 4:**
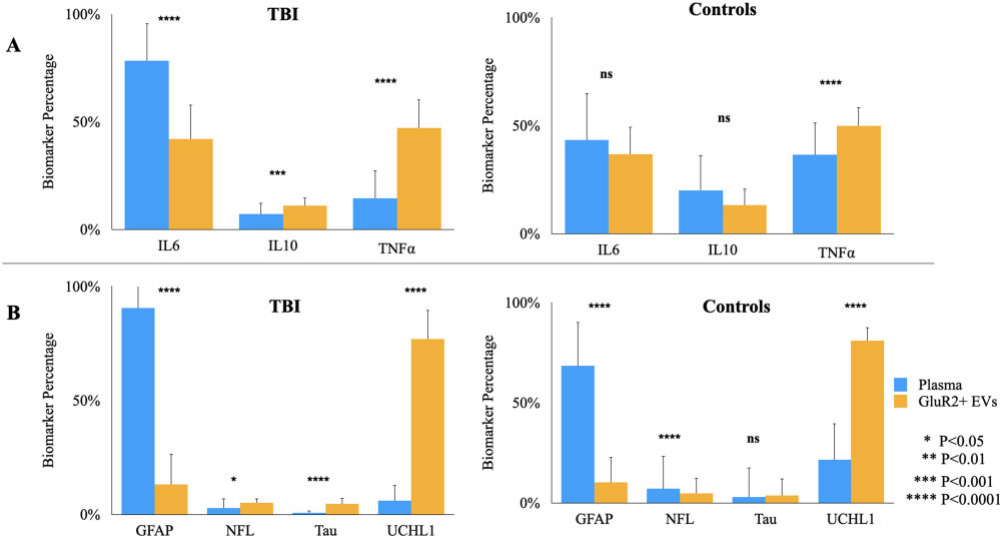
**Plasma- and brain-derived EVs possess distinct protein composition that are each altered by TBI.** Mean levels of each biomarker were totalled across individuals to determine the relative percentage of each (**A**) cytokine and (**B**) brain-derived protein in plasma and GluR2+ EVs. Error bars represent SD calculated by propagation of uncertainty. *T*-tests were performed to assess statistically significant differences in biomarker levels across compartments.

Until this point, we analysed the performance of single proteins, regardless of its originating in plasma or GluR2+ EVs, to best discriminate between mTBI and control samples. However, simply combining the best individually high performing biomarkers would potentially overlook combinations of biomarkers that would better predict the presence/absence of mTBI. To evaluate distinctions in biomarker information across plasma and GluR2+ EV compartments, we first assessed the distribution of biomarkers in each group. In the mTBI group, plasma and GluR2+ EVs displayed significantly different proportions of all measured cytokines (*P* < 0.001 across all cytokines; Fig. 4A). Specifically, the relative abundance of IL6 was significantly elevated in plasma compared to GluR2+ EVs. Conversely, GluR2+ EVs contain significantly higher proportions of both IL10 and TNFα than plasma. The distribution of cytokines is also more balanced in GluR2+ EVs, with IL10 and TNFα making up similar proportions, while in plasma, cytokine distribution is skewed with the relative abundance of IL6 dwarfing that of IL10 and TNFα by 11- and 5-fold respectively. In contrast to the mTBI group, the control group displayed no significant differences in proportions of IL6 or IL10 across the two compartments (*P* > 0.05). In this group, only TNFα abundance differed in plasma and GluR2+ EVs, showing a significant increase in the latter (*P* < 0.0001). Lastly, in this group, distribution of the three cytokines exhibits balance in both compartments, each having similar proportions of IL6 and TNFα.

Like the cytokines, our analysis also revealed differences between plasma and GluR2+ EV distributions of the four brain-derived proteins (Fig 4B). For both mTBI and control subjects, abundance of three of the four (GFAP, NFL and UCHL1) display significant differences in plasma compared to GluR2+ EVs, and Tau abundance is significantly elevated in GluR2+ EVs in the mTBI group (*P* < 0.05 across all brain-derived proteins; Fig. 4B). In both groups, the distributions of these proteins are uniquely skewed in each compartment; in plasma, GFAP abounds (fold increases of 31, 136 and 15 relative to NFL, Tau and UCHL1 respectively for mTBI group; fold increases of 10, 24 and 3 relative to NFL, Tau and UCHL1 respectively for controls) while GluR2+ EVs are dominated by UCHL1 (fold increases of 6, 15 and 17 compared to GFAP, NFL and Tau respectively for mTBI group; fold increases of 8, 16 and 22 relative to GFAP, NFL and Tau respectively for controls).

### Biomarker levels correlate more within than across plasma- and brain-derived EV compartments

**Figure 5:**
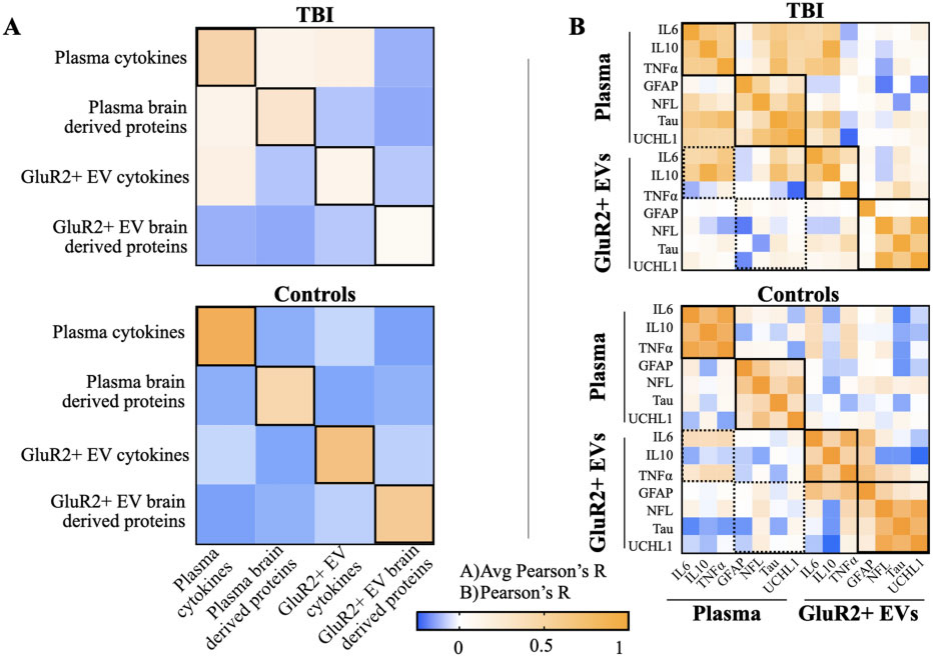
**Biomarker levels are uncorrelated across plasma and brain-derived EV compartments.** Pearson’s correlation coefficients calculated for all possible combination of biomarkers. (**A**) Average *R* for each biomarker type (cytokines or brain-derived markers) and for each compartment (plasma or GluR2+ EVs) were plotted in a heat map matrix for TBI patients and controls. Solid boxes indicate average *R* for each biomarker type-compartment combination. (**B**) *R* for each biomarker comparison was plotted into heat map matrices for both TBI patients and controls. Solid boxes indicate *R* for individual biomarkers of the same type (cytokines or brain-derived proteins) within each compartment. Dashed boxes indicate *R* values for biomarkers of the same type, but of different compartments.

To develop a combinatorial method for discriminating TBI from control subjects, each biomarker should hold the potential to contribute unique information about each patient’s TBI. To assess this, we calculated three sets of correlation values for each biomarker category (cytokines or brain-derived proteins): correlations within plasma, within the GluR2+ EVs, and across these two compartments (Fig. 5A).

We found levels of cytokines (IL6, IL10 and TNFα) and brain-derived proteins (GFAP, NFL, Tau and UCHL1) correlated more with each other within the same compartment than between the plasma and EVs compartments (Fig. 5B). For mTBI subjects, cytokine levels are most correlated within the plasma (avg. Pearson’s *R* = 0.61). In comparison, cytokine correlations within the EV compartment (avg Pearson’s *R* = 0.38) and across plasma and EVs (avg Pearson’s *R* = 0.41) were similar. Levels of brain-derived biomarkers are more correlated within plasma (avg Pearson’s *R* = 0.50) and within GluR2+ EVs (avg Pearson’s *R* = 0.35) than across plasma and GluR2+ EV compartments (avg Pearson’s *R* = 0.0065). As with the mTBI group, cytokine levels are most correlated within plasma (avg Pearson’s *R* = 0.80) in the control group. In contrast to the mTBI group, cytokine levels are also more correlated within EVs (avg. Pearson’s *R* = 0.57) than across plasma and EVs (avg. Pearson’s *R*=0.20). Like the mTBI group, pools of brain-derived biomarkers are more distinct: levels of these proteins are most correlated in EVs and within the plasma compartment, and least correlated across compartments (avg. Pearson’s *R* = 0.55, 0.42, 0.0065, respectively). Our results showed that different groups of biomarkers had very little correlation across compartments though levels correlated within each group.

Given the independence of information collected from plasma and plasma-derived EVs, we next assessed whether we could develop a machine learning-based classifier of TBI using the complimentary biomarker information contained within each. By first separating out a group of samples to train our model and using a separate group to test its accuracy, we found the combination of information from plasma-derived EVs and plasma showed high accuracy (AUC = 0.913, Accuracy = 0.825). However, this combination of measures was only marginally better than a single measure of plasma GFAP or IL-6 (Supplementary Fig. 3) and did slightly better in prediction of injury when compared to combination of measures from plasma. However, removing a measure of Tau in the EVs significantly worsened the predictive power of the panel, implying the importance of including an EV-based measure in a combined diagnostic (Supplementary Fig. 3). While the sample size in this study is not sufficient to fully develop and evaluate a machine learning classifier, these results suggest its promise in future clinical studies.

## Discussion

Our study demonstrates that circulating brain-derived EVs and plasma represent two distinct reservoirs of molecular information, the composition of each differentially altered by mTBI. We combined two technologies—TENPO that can specifically enrich for GluR2+ EVs from plasma, and digital ELISA that can measure multiple protein biomarkers with 100–1000× better sensitivity than conventional ELISA—to address the challenges of accurately detecting levels of biomarkers that often circulate at levels too low to detect with conventional technologies. We then demonstrate that algorithmically combining information from each biomarker compartment accurately classifies mTBI (AUC = 0.92, Accuracy = 0.885). The combined use of biomarkers of specific TBI pathologies analysed in the context of distinct biofluid environments from separate cellular pools is a promising approach for developing a more comprehensive assessment of the state of the injured and recovering brain.

We began our analysis with measuring circulating levels of brain-derived proteins GFAP, NFL, Tau and UCHL1, which demonstrated predictive power as individual biomarkers similar to past studies of these biomarkers in mTBI.^19^^,^^45^ Since a sizable proportion of mTBI subjects in this study (57%) sustained brain pathology observable through CT scan, the significant elevations in plasma levels of UCHL1 and NFL (*P* < 0.0001 for each) were expected.^19^ As mTBI results in few degenerating neurons,^46^ it is not surprising that we did not detect significant elevations in Tau after mTBI. In contrast, reactive gliosis can be observed throughout the brain even after mild injury,^47^ and as expected we found plasma GFAP the most robust single biomarker to discriminate mTBI subjects from controls (AUC = 0.89). However, we observed plasma GFAP levels were significantly elevated in orthopaedic-injured controls and controls over 51 years (ANOVA; *P* < 0.046 and *P* < 0.0001, respectively) compared to the total control plasma GFAP mean. This finding, combined with observations that GFAP is released from other cell types of the body,^48^ may complicate GFAP’s specificity to brain injury, especially if it is used in isolation in polytrauma cases. We also observed significant elevations in plasma levels of IL6, IL10 and TNFα (*P* < 0.001 across all cytokines), and indeed, plasma IL6 followed directly behind plasma GFAP in discriminating mTBI (AUC = 0.86). But the broad role that cytokines play in mediating systemic trauma,^49^ immune challenges^50^^,^^51^ and other neurological disorders^52^ may limit the specificity of these biomarkers in plasma.

In our assessment of mTBI, we also included brain-derived EVs expressing GluR2, an appealing alternative to co-opting the circulating neurodegenerative markers typically associated with moderate-to-severe TBI for mTBI diagnostics. On their own, the proteins in brain-derived EVs performed no better than those in plasma as individual biomarkers, despite the IL6 and GFAP elevations observed in this compartment relative to controls (*P* < 0.01 and *P* < 0.001, respectively). However, it was intriguing to see that protein concentration of the same biomarkers across the two compartments did not correlate with each other. One potential explanation for this result is that plasma levels of some biomarkers appeared from active degeneration processes in a small population of cells, while the exosome derived measurements originate from a large population of largely intact neurons and glia responding to the mild mechanical trauma. Other studies of EV-based biomarkers of TBI have similarly observed differences in EV-contained and plasma molecular cargo. In a study measuring time-dependent changes in protein biomarkers within the total circulating EV population and plasma, investigators found no correlation between the two compartments out to 5 days after injury.^53^

Although studies on EVs and their contents is only emerging, the broader sampling of EV signatures from cells that do not later degenerate provides a new opportunity for understanding the consequences and recovery processes of mild trauma to the brain. With the broad disruption in blood–brain barrier integrity that occurs after mTBI,^54^ it is possible that plasma activates pathologic cascades in neurons and glia that do not later degenerate, resulting in a cellular population that largely outnumbers actively dying or degenerating ones in mTBI that are not assessed with traditional plasma biomarkers. In experimental models of concussion and in clinical studies, degenerative changes can occur days to months following the initial mild injury, and can be further complicated by repeated, periodic opening of the blood–brain barrier.^55–58^ These primed or activated neurons and glia undergo subtler forms of cellular damage or distress as they constitutively secrete exosomes, potentially as a mechanism for clearing cellular debris as they recover. We observed that GluR2+ EVs contain the same inflammatory cytokines and markers of cell damage expressed by lesioned cells.^59^ Interestingly, we found UCHL1—a deubiquitinating enzyme—dominated the EV pool of brain-derived proteins (Fig. 4). As UCHL1 plays a neuroprotective role in brain injury by degrading reactive lipids and misfolded proteins,^60^^,^^61^ the high relative abundance of this protein in GluR2+ EVs in relation to the other measured brain-derived proteins suggests GluR2+ EVs may serve as a protein clearing system for the damaged or distressed cells of the brain, a role for EVs already demonstrated for other EV populations in other contexts and cell types.^62^ Thus, our machine learning approach combines molecular information from three different categories: markers from a small number of severely damaged, degenerating cells (plasma brain-derived markers), markers of broad-scale inflammation, and markers originating from a potentially less-damaged population of brain cells (brain-derived EVs). By investigating what downstream molecular targets GluR2+ EV-packaged UCHL1 interacts with, such as other components of the ubiquitin ligase system and potential degradation targets, we can broaden our understanding of the role the GluR2+ EV population plays in TBI pathology, and our potential pool of EV-associated biomarkers.

Though we demonstrate the orthogonality of molecular information in plasma and brain-derived EVs, the machine panel we devised by algorithmically combining molecular information across the two compartments did not significantly benefit mTBI diagnosis when compared to the performance of plasma neurodegenerative markers alone. Our analysis of brain-derived EVs was limited to those expressing GluR2+, and by expanding our approach to mTBI biomarker development—from broadening the EV subtypes and cargo that we isolate to surveil a more comprehensive set of cells affected by TBI, to advancing the technologies with which we measure and analyse this complex information—we can improve our ability to diagnose mTBI, and to extend this approach to monitor mTBI outcome and identify accurate treatment strategies. Since this work we have extensively optimized the TENPO protocol, incorporating sequential wash steps following vesicle capture which greatly reduce background relative to relying on the chip’s small dead volume to promote removal of unbound material.^39^ Additionally, while lysis with 0.1% SDS using RIPA buffer resulted in lower protein concentration than lysing with 1% SDS (Supplementary Fig. 2), it is possible that this resulted in some protein denaturing. Since this work, we have improved lysis conditions to maximize protein yield and efficacy of biomarker detection. We now use phosphatase/protease inhibitor cocktail and are investigating non-denaturing reagents, both of which have been used in other recently published SIMOA studies of exosome protein expression.^35^^,^^53^

We are also exploring capturing circulating EVs derived from multiple brain cell types (neurons, astrocytes, microglia, etc.), which, when combined with advancements in downstream EV cargo analysis, maximizes the molecular information at our disposal for these goals. Multianalyte approaches to disease diagnosis have already shown promise in other fields, resulting in higher accuracy in early detection and staging of cancer.^63–65^ For mTBI, as technology for isolating EVs from different populations of distressed-but-not-dying brain cells evolves, we may improve our ability to identify pathologies like gliosis and brain endothelial cell dysfunction as they occur across individual patients to better ‘grade’ the TBI. Though we used digital ELISA in this study, a platform incompatible with point-of-care diagnostics, widespread efforts to scale down these assays into portable platforms makes accessibility and clinical use of molecular diagnostic more achievable.^66^ Our previous work also points to the promise of miRNA cargo for a more open-ended TBI assessment than is provided by known protein biomarkers, but combining EV protein cargo information with amplifiable EV-associated miRNA results in a wealth of potential opportunities to develop more accurate mTBI characterization. Advanced approaches to data analysis such as machine learning coupled with improved understanding of the pathologic roles brain-derived EVs play in mTBI progression broadens our potential to combine this wealth of information into meaningful molecular signatures to monitor and intervene in this insidious neurologic condition.

## Supplementary material

Supplementary material is available at *Brain Communications* online.

## Supplementary Material

fcab151_Supplementary_Data

## Abbreviations

AUC =: area under the receiver operating characteristic curve
EV =: extracellular vesicles
GFAP =: glial fibrillary acid protein
GluR2 =: glutamate ionotropic receptor AMPA type subunit 2
IL6 =: interleukin-6
IL10 =: interleukin-10
mTBI =: mild traumatic brain injury
NFL =: neurofilament light
ROC =: receiver operating characteristic
SIMOA =: single-molecule array
TENPO =: track etched magnetic nanopore
TNFα =: tumour necrosis factor alpha
UCHL1 =: ubiquitin C-terminal hydrolase L1
