# Supplementary material for: Extracellular vesicles as distinct biomarker reservoirs for mild traumatic brain injury diagnosis
Source: Brain Commun. 2021 Jul 8;3(3):fcab151. doi: 10.1093/braincomms/fcab151 (PMC8491985; doi:10.1093/braincomms/fcab151)
Supplement: fcab151_Supplementary_Data [file fcab151_supplementary_data.pdf]

SUPPLEMENTARY MATERIALS

Supplemental figure 1: Scanning electron microscopy (SEM) images of TENPO-captured EVs at A) 50,000x and B) 40,000x.

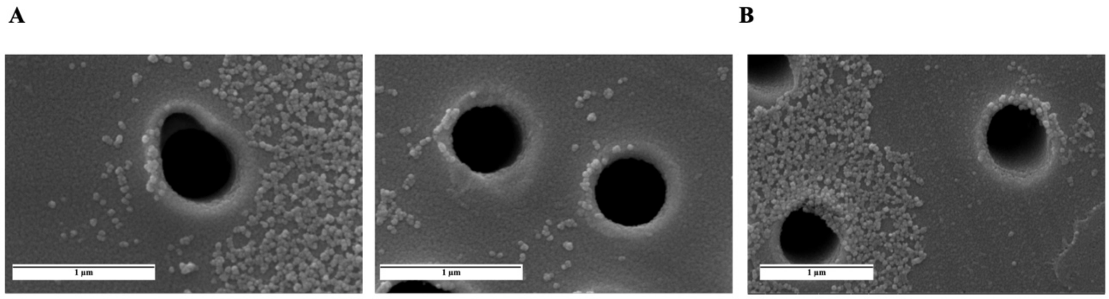

Supplemental figure 2: Cytokine 3Plex levels from EVs derived from *N*=1 healthy control under different lysis conditions. Sample lysed with 1% SDS was diluted 1:10 before taking the measurements.

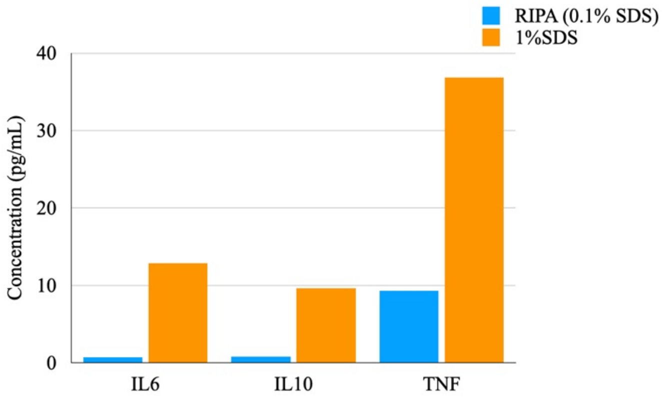

Supplemental Figure 3: A multi-analyte biomarker panel can accurately classify mTBI. **A)** Performance of machine learning ensemble classifier using biomarkers from both GluR2+ EV and plasma compartments. **B)** Machine learning panel performance on user blind test subjects. **C)** Comparison of machine learning panel performance with single biomarker performance. *indicates z score >1.96; p<0.05 relative to machine learning (ML) panel using paired t Test while considering AUCs correlation induced by the nature of data. Error bars for single biomarker AUCs represent standard deviation of biomarker performance across 10 trials of random subsets of the dataset (90% of the total sample size) with replacement using bootstrapping. **D)** Performance of panels consisting of biomarkers from plasma (plasma Neuro4Plex, plasma Cytokine3Plex) or GluR2+ EVs (GluR2+ Neuro4Plex, GluR2+ Cytokine3Plex) alone, and panels of 2,3,4, or all 5 of the biomarkers of the machine learning panel compared to the performance of the full multi-compartment machine learning panel. * indicates z score >1.96 relative to ML panel using paired t Test while considering AUCs correlation. **E)** AUC of panels consisting of biomarkers from plasma (plasma Neuro4Plex, plasma Cytokine3Plex) or GluR2+ EVs (GluR2+ Neuro4Plex, GluR2+ Cytokine3Plex) alone, and panels of 2,3,4, or all 5 of the biomarkers of the machine learning panel compared to the performance of the full multi-compartment machine learning panel. **F)** Change in machine learning model AUC following removal of markers from the panel. White boxes indicate the marker(s) removed. Error bars indicate standard error after 50 trials.

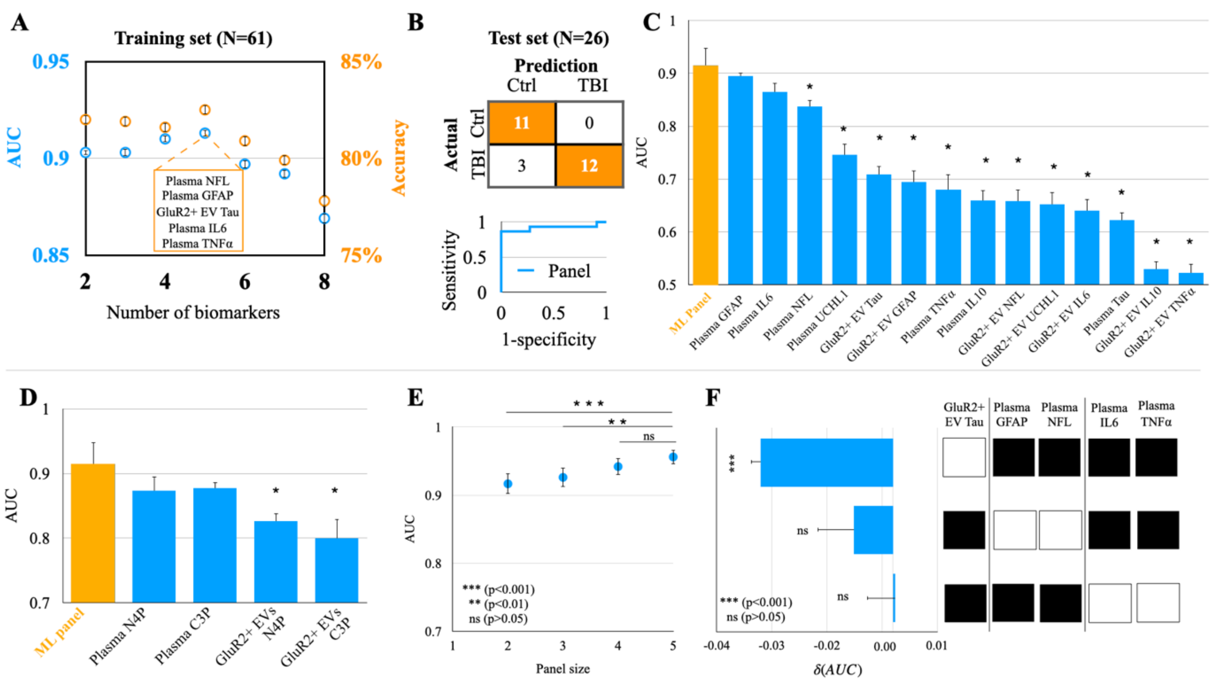

Notes on the development of a machine learning classifier of mTBI.

To develop a machine learning based classifier of TBI with GluR2+EV and plasma biomarker levels, we first applied Least Absolute Shrinkage and Selection Operator (LASSO) on the training set of data (*n*=61) and determined the best performing panel for discriminating TBI from controls (AUC=0.913, Accuracy=0.825, Fig.S3A). We then applied it to an independent, blinded test set of 26 subjects (AUC=0.92, Accuracy=0.885, Fig.S3B). Though the machine learning panel led to a significant improvement in discriminating mTBI patients from controls (AUC) compared to most of the individual biomarkers from both plasma and GluR2+ EVs ($z > 1.96$; $p<0.05$ Fig.S3C), it did not result in a significant improvement when compared to AUCs obtained from plasma GFAP ($z = 0.71$; $p=0.47$) or plasma IL6 ($z=1.4$; $p=0.16$) when tested on the consolidated dataset (training + test). Similarly, though the machine learning panel exhibited the highest AUC,

the added benefit did not reach significance when compared with the best-performing single compartment panels ($z = 1.11$; $p=0.27$ for plasma brain derived proteins; $z = 1.1$; $p=0.26$ for plasma cytokines; Fig.S3D). However, the machine learning panel did outperform the best performing panels with fewer biomarkers derived from the original panel, i.e. a panel of plasma NFL and GFAP ($p<0.001$) and a panel of plasma NFL, GFAP, and GluR2+ EV Tau ($p<0.01$) when tested across the combined training and test set (Fig. S3E). Lastly, we observed a significant decline in the model's performance upon removing GluR2+ EV Tau from the panel (Fig.S3F; $p<0.001$). Neither the plasma brain derived markers (GFAP and NFL) nor cytokines (IL6 and TNFα) affected the panel's performance when removed.

Supplementary table 1: P values for ANOVA calculation of effect of injury type on plasma biomarker levels.

Biomarker	P Value
IL6	0.78
IL10	0.74
TNFα	0.73
GFAP	0.96
NFL	0.23
Tau	0.52
UCHL1	0.29

Results obtained using log values and corrected for multiple comparisons using Tukey's post-hoc analysis. Groups assessed include TBI assault (*n*=8), TBI road traffic (*n*=15), and TBI fall (*n*=21). TBI "other" group excluded for insufficient *n* (*n*=3).

Supplementary table 2: P values for student's t test calculation of effect of control type on plasma biomarker levels.

Biomarker	P Value
IL6	0.015*
IL10	0.59
TNFα	0.23
GFAP	0.74
NFL	0.39
Tau	0.35
UCHL1	0.73

Orthopedic control (*n*=7), healthy control (*n*=39); *indicates $p<0.05$.

Supplementary Table 3: P values for ANOVA calculation of effect of injury type on GluR2+ EV biomarker levels.

Biomarker	P value
IL6	0.97
IL10	0.59
TNFα	0.89
GFAP	0.70
NFL	0.38
Tau	0.48

UCHL1	0.33

Results obtained using log values and corrected for multiple comparisons using Tukey's post-hoc analysis. Groups assessed include TBI assault (*n*=8), TBI road traffic (*n*=15), and TBI fall (*n*=21). TBI "other" group excluded for insufficient *n* (*n*=3).

Supplementary Table 4: P values for student's t test calculation of effect of control type on GluR2+EV biomarker levels.

**Biomarker**	**Control orthopedic**
IL6	0.72
IL10	0.069
TNFα	0.67
GFAP	0.24
NFL	0.62
Tau	0.14
UCHL1	0.13

Orthopedic control (*n*=7), healthy control (*n*=39).

Supplementary Table 5: R^2 values for calculation of effect of age on TBI patient and control log plasma biomarker levels.

**Biomarker**	**TBI**	**Control**
N	47	46
IL6	0.00011	0.094
IL10	0.0090	0.0032
TNFα	0.62	0.083
GFAP	0.40	0.35

NFL	0.62	0.20
Tau	0.0017	1.3E-005
UCHL1	0.028	0.0014

Supplementary Table 6: R^2 values for calculation of effect of age on TBI patient and control GluR2+ EV log biomarker levels.

**Biomarker**	**TBI**	**Control**
N	47	46
IL6	0.023	0.028
IL10	0.097	0.035
TNFα	0.00081	0.015
GFAP	0.014	0.023
NFL	0.050	0.035
Tau	0.087	0.0059
UCHL1	0.049	0.011
